# TGF-β blockade depletes T regulatory cells from metastatic pancreatic tumors in a vaccine dependent manner

**DOI:** 10.18632/oncotarget.5656

**Published:** 2015-10-15

**Authors:** Kevin C. Soares, Agnieszka A. Rucki, Victoria Kim, Kelly Foley, Sara Solt, Christopher L. Wolfgang, Elizabeth M. Jaffee, Lei Zheng

**Affiliations:** ^1^ Department of Oncology, Johns Hopkins University School of Medicine, Baltimore, MD, USA; ^2^ Department of Pathology, Johns Hopkins University School of Medicine, Baltimore, MD, USA; ^3^ Department of Surgery, Johns Hopkins University School of Medicine, Baltimore, MD, USA; ^4^ The Sidney Kimmel Cancer Center, Johns Hopkins University School of Medicine, Baltimore, MD, USA; ^5^ The Skip Viragh Center for Pancreatic Cancer Research and Clinical Care, Johns Hopkins University School of Medicine, Baltimore, MD, USA; ^6^ The Sol Goldman Pancreatic Cancer Center, Johns Hopkins University School of Medicine, Baltimore, MD, USA

**Keywords:** vaccine, pancreatic cancer, immunotherapy, TGF-beta, regulatory T cells

## Abstract

Our neoadjuvant clinical trial of a GM-CSF secreting allogeneic pancreas tumor vaccine (GVAX) revealed the development of tertiary lymphoid aggregates (TLAs) within the pancreatic ductal adenocarcinoma (PDA) tumor microenvironment 2 weeks after GVAX treatment. Microarray studies revealed that multiple components of the TGF-β pathway were suppressed in TLAs from patients who survived greater than 3 years and who demonstrated vaccine-enhanced mesothelin-specific T cell responses. We tested the hypothesis that combining GVAX with TGF-β inhibitors will improve the anti-tumor immune response of vaccine therapy. In a metastatic murine model of pancreatic cancer, combination therapy with GVAX vaccine and a TGF-β blocking antibody improved the cure rate of PDA-bearing mice. TGF-β blockade in combination with GVAX significantly increased the infiltration of effector CD8^+^ T lymphocytes, specifically anti-tumor-specific IFN-γ producing CD8^+^ T cells, when compared to monotherapy controls (all *p* < 0.05). TGF-β blockade alone did not deplete T regulatory cells (Tregs), but when give in combination with GVAX, GVAX induced intratumoral Tregs were depleted. Therefore, our PDA preclinical model demonstrates a survival advantage in mice treated with an anti-TGF-β antibody combined with GVAX therapy and provides strong rational for testing this combinational therapy in clinical trials.

## INTRODUCTION

Despite exciting breakthroughs in cancer treatment with novel immunotherapies, pancreatic ductal adenocarcinoma (PDA) remains highly resistant to these agents. This is due to immune tolerance mechanisms initiated early in the development of PDA [1]. These mechanisms include early infiltration of the tumor microenvironment (TME) with a variety of immunosuppressive cells including tumor-associated macrophages (TAMs), myeloid derived suppressive cells (MDSCs), and regulatory T cells (Tregs) [1]. Additionally, antigen experienced effector T cells (Teffs) are scarce [2]. While a number of studies have demonstrated the promise of vaccine-based immunotherapy for pancreatic cancer, effective strategies targeting the immunosuppressive cells are lacking [3–5].

Various types of tumors, including PDA, produce TGF-β and exploit this mechanism to evade immune attack via binding to its receptor [16]. There are three isotypes of TGF-β, TGF-β 1, 2, and 3. The TGF-β receptor (TGF-βR) is a heterodimer formed by TGF-βRI and TGF-βRII. The overexpression of TGF-β by tumor cells suppresses T cell responses through a variety of TGF-β signaling pathways. TGF-β has also been shown to be a crucial signal that regulates Tregs. Published studies suggested that TGF-β induces the expression of the transcription factor forkhead box 3 (FoxP3), which is a master regulator in the development and differentiation of CD4^+^CD25^+^ Tregs [17–19]. These regulatory T cells then secrete TGF-β and other inhibitory cytokines to suppress CD8^+^ T cell killing of tumor cells [19].

Several studies examined the feasibility of enhancing anti-tumor immune responses through the inhibition of Treg activity by CD25 cell surface marker blockade [6– 8]. Although depletion of Tregs via CD25 blockade leads to enhanced immunity in tumor models, removal of T cells expressing CD25 also leads to the removal of Teffs, which express CD25 after activation. Additionally, this therapy currently lacks translational applicability since a human antibody equivalent that can effectively deplete human CD25^+^ T cells is unavailable. Tregs constitutively express FoxP3, cytotoxic T lymphocyte-associated protein 4 (CTLA-4), and glucocorticoid-induced TNF receptor family-related protein (GITR) [9]. These factors are believed to be crucial for the suppressive function of Tregs. Therefore, depleting Tregs by targeting cell markers such as CTLA-4 were studied. While clinical efficacy has been demonstrated with anti-CTLA-4 antibodies, a number of severe autoimmune side effects have been observed, including inflammatory bowel pathology and hyphophysitis [11]. Biologically, blockade of CTLA-4 may not be sufficient given that Tregs from CTLA-4 knockout mice are still capable of suppressing the immune response [12]. More recently, suppressing Treg cell activity through GITR has been studied in tumor models [13]. However, the human GITR blockade antibody is still in the early phase clinical trials. Interestingly, low-dose cyclophosphamide not only decreases cell numbers of Tregs but leads to decreased functionality of Tregs [15]. However, cyclophosphamide also depletes Teffs. Thus, optimal Treg targeting agents are lacking and are urgently needed to improve the efficacy of immunotherapies for PDA treatment.

Blocking TGF-β or its receptors has been shown to have anti-cancer activities in preclinical cancer models [20–22]. As mentioned above, TGF-β appears to be a target for blocking Tregs. However, TGF-β blocking agents have not yet been shown to target Tregs. The clinical development of TGF-β blocking agents is still challenging due to the lack of significant efficacy data for single agent TGF-β targeting therapies; however, recent early phase clinical trials using TGF-β blocking agents in certain gastrointestinal cancers have shown promising results [23].

Our group recently conducted a novel neo-adjuvant and adjuvant study designed to evaluate post-immunotherapy changes within the TME of primary pancreatic tumors following treatment with our pancreatic cancer GVAX vaccine, given either alone or with immune modulating doses of cyclophosphamide [3]. With the same pancreatic cancer vaccine, it was previously reported that low dose cyclophosphamide enhanced higher avidity T cell responses that were associated with longer progression free survival in patients [24]. The aforementioned neoadjuvant vaccine study provided the opportunity to dissect the PDA TME in the wholly resected tumors. Pathological examination of tumor tissue resected just two weeks following vaccination identified the formation of novel immunotherapy-induced tertiary lymphoid aggregates (TLAs), an organized lymphoid structure that was not observed in tumors resected from unvaccinated patients. Gene microarray analysis of micro dissected vaccine-induced lymphoid aggregates identified gene expression in the TGF-β pathway, which correlated with improved patient outcomes. Most genes in the TGF-β pathway, including multiple TGF-β isotypes and TGF-βR subunits, were downregulated in lymphoid aggregates from patients who survived more than 3 years, in patients who demonstrated vaccine-enhanced mesothelin-specific T cell responses, and in patients with increased Teff/Treg (CD8/Foxp3) ratios within their tumors [3]. These results suggested that targeting the TGF-β pathway might further enhance antitumor immune response induced by vaccine therapy. Therefore, in this study, we tested this hypothesis in preclinical models of PDA and subsequently demonstrated a significant effect of TGF-β blocking antibodies on Tregs in a vaccine dependent manner. Our study supports the clinical evaluation of TGF-β blocking agents as combinational immunotherapy with cancer vaccines.

## RESULTS

### TGF-β blockade in combination with GVAX improves the cure rate of metastatic PDA in murine tumor models

**Figure 1:**
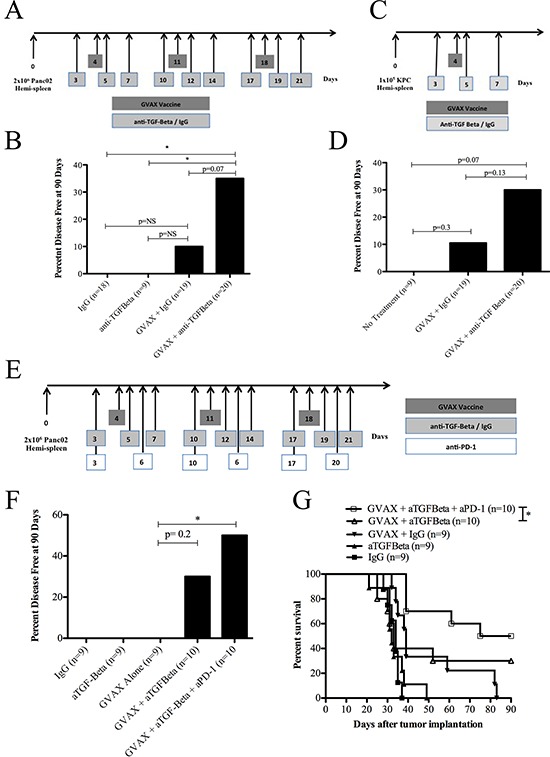
Combination therapy with GVAX and αTGF-β blockade improves clinical outcomes in a PDA mouse model **A.** Schema of tumor implantation by the hemispleen procedure and treatment with GVAX and αTGF-β blockade as indicated. C57Bl/6 mice were challenged on day 0 with 2 × 10^6^ Panc02 tumor cells followed by administration of irradiated whole cell GM-CSF Panc02 GVAX on days 4, 11, 18. αTGF-β or IgG was administered IP at 100 ug three times weekly for 3 weeks starting on day 3. **B.** The percentage of mice that remained disease free at day 90 following Panc02 tumor implantation with GVAX and/or αTGF-β/IgG. **C.** Schema of an additional PDA tumor implantation model consisting of 1 × 10^5^ KPC tumor cells injected on day 0 via hemispleen technique in C57Bl/6 mice. A single dose of irradiated GVAX was administered on day 4. αTGF-β or IgG was administered on days 3, 5 and 7 at 100 ug IP. **D.** The percentage of mice remaining disease free at day 90 following KPC tumor implantation with GVAX and/or αTGF-β/IgG. **E.** Schema of tumor implantation by the hemispleen procedure and treatment with GVAX, TGF-β blockade and αPD-1 as indicated. C57Bl/6 mice were challenged on day 0 with 2 × 10^6^ Panc02 tumor cells followed by administration of irradiated whole cell GM-CSF Panc02 GVAX on days 4, 11 and 18. αTGF-β or IgG was administered IP at 100 ug three times weekly for 3 weeks starting on day 3. αPD-1 was administered IP at 100 ug twice weekly for 3 weeks starting on day 3. **F.** The percentage of mice that remained disease free at day 90 following Panc02 tumor implantation with GVAX and/or αTGF-β/IgG and/or αPD-1. **G.** Kaplan-Meier survival curves of mice that were implanted with Panc02 tumor cells via hemispleen technique and treated with different combinations of Panc02 GVAX, αTGF-β, IgG and/or αPD-1. Data are represented as results obtained from experiments with 8 to 10 mice per group, pooled and repeated at least twice. NS, not significant; *p < 0.05. PDA, pancreatic ductal adenocarcinoma. IP, intraperitoneal.

We examined whether combining GVAX with a monoclonal pan-TGF-β neutralizing antibody, which blocks all 3 of the TGF-β subtypes (αTGF-β), TGF-β 1,2, & 3, could improve the anti-tumor activity of GVAX in two hepatic metastatic PDA models. The metastatic PDA tumor model was established by hemispleen injection of Panc02 or KPC tumor cells [25]. αTGF-β or IgG isotype controls were administered either as a monotherapy or in combination with GVAX (Figure 1A). For the Panc02 model, αTGF-β or IgG isotype control was administered 3 times per week for 3 weeks while GVAX was administered once weekly for three weeks on days 4, 11 and 18 post tumor inoculation. The KPC model treatment regimen consisted of αTGF-β or IgG controls dosed on days 3, 5, and 7 post tumor inoculation with a single GVAX vaccination on day 4.

Mice in the liver metastasis model bear a high burden of malignant cells. Vaccine-based monotherapy has been shown to cure only approximately 10% of the mice. αTGF-β therapy alone did not cure any mice in this model. Combinatorial GVAX and αTGF-β therapy significantly improved the cure rate of mice compared to IgG control treatment and αTGF-β monotherapy (35% vs. 0%, *p* < 0.05) in the Panc02 tumor cell hemispleen model (Figure 1B). When compared with GVAX plus IgG, the possibility of combinatorial GVAX and αTGF-β having improved cure rates cannot be excluded (35% vs. 10.5%, *p* = 0.07). Similar experiments were performed to investigate this effect in the KPC tumor cell hemispleen PDA model (Figure 1C). Again, the possibility of the combination of GVAX and αTGF-β having improved cure rates when compared to GVAX plus IgG (30% vs. 11%, *p* = 0.13) cannot be excluded (Figure 1D). These data suggest that, although TGF-β blockade itself was not found to have an antitumor activity in the tumor model tested here, TGF-β blockade is able to enhance the antitumor activity of GVAX.

TGF-β blockade increased the cure rate with GVAX to approximately 30%, suggesting that other immunosuppressive pathways need to be targeted simultaneously. We have previously showed that targeting the PD-1 pathway in combination with GVAX can enhance the cure rate to approximately 40% [26]. Therefore, we tested the combination of αTGF-β and αPD-1 antibody with GVAX (Figure 1E) and found that both blocking agents together can increase the cure rate with GVAX to 50% and significantly improve median overall survival versus GVAX and αTGF-β therapy alone (33 days vs. 82.5 days, *p* < 0.05) (Figure 1F and 1G). This result suggests that TGF-β targets a non-PD-1 pathway, and its blockade can enhance PD-1 blocking activity.

### TGF-β blockade reduces tregs in the PDA TME in a GVAX therapy dependent manner

**Figure 2:**
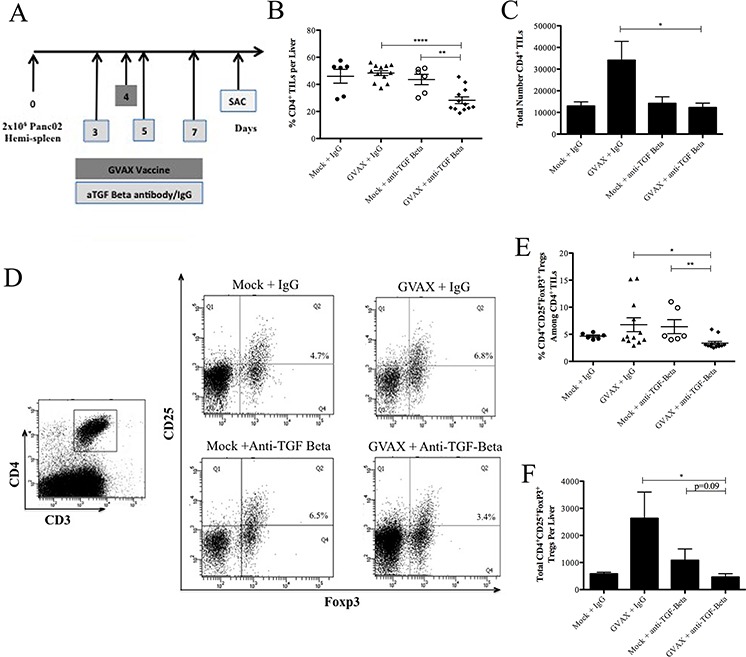
Combination therapy with GVAX and αTGF-β decreases CD4^+^ T cell presence including Tregs in PDA **A.** Schema of immune analysis following tumor implantation by the hemispleen procedure and treatment with αTGF-β or IgG (100 ug IP) on days 3, 5, 7 and GVAX on day 4. **B.** The percentage of CD4^+^ T cells among total lymphocytes and **C.** the total number of CD4^+^ TILs after Panc02 hemispleen and the indicated therapy. **D.** Fluorescence-activated cell sorting (FACS) cytometry gating schema and density plot for CD4^+^CD25^+^Foxp3^+^ Tregs among TILs. Histogram showing **E.** the percentage of Tregs amongst TILs and **F.** the total number of Tregs in the PDA TME after the indicated therapy. Each experiment consisted of 3 or 6 mice per group, pooled and analyzed individually in duplicate. Data represent mean ± SEM from one experiment repeated at least twice. *p < 0.05, ^**^p < 0.01. TILs, tumor infiltrating lymphocytes. PDA, pancreatic ductal adenocarcinoma. TME, tumor microenvironment. Tregs, regulatory T cells. IP, intraperitoneal

Our prior analysis of dissected human PDA lymphoid aggregates showed that TGF-β signaling pathways were downregulated and intratumoral Tregs were decreased in vaccinated patients who had longer survival [3]. We therefore examined whether there is an improved antitumor efficacy of TGF-β blockade in combination with vaccine therapy. We first evaluated the CD4^+^ T cell population within the TME of metastatic Panc02 tumor bearing mice. Tumor-bearing mice were treated with either αTGF-β or IgG control on days 3, 5, and 7. GVAX was administered once on day 4 (Figure 2A). On day 10, the livers were harvested for fluorescence-activated cell sorting (FACS) analysis of liver infiltrating lymphocytes (TIL).

The percentages of CD4^+^ TILs among all TILs were significantly lower in the combinatorial treatment group compared to GVAX monotherapy (28.3% vs. 48.4%, *p* < 0.0001) and αTGF-β monotherapy (28.3% vs. 48.6%, *p* < 0.01) (Figure 2B). The total number of CD4^+^ TIL was significantly lower in the combinatorial GVAX αTGF-β group compared to the GVAX monotherapy (12,211 vs. 34,067 CD4^+^ T cells per liver, *p* < 0.05) (Figure 2C). When looking specifically at CD4^+^CD25^+^Foxp3^+^ Tregs (Figure 2D), GVAX therapy induced intratumoral Tregs, suggesting that Tregs confers a checkpoint for vaccine-induced T cell responses. However, the combinatorial group had a significantly lower percentage of Tregs among all TILs as well as a reduction in the total absolute number of Tregs within the TME when compared to GVAX monotherapy (3.4% vs. 6.8%, *p* < 0.05; 461 vs. 2,629 Tregs per liver, respectively, *p* < 0.05) (Figure 2E and 2F, respectively). By contrast, αTGF-β monotherapy did not decrease either the percentage of Tregs or its absolute number within the TME compared to the IgG control. Notably, combinatorial GVAX and αTGF-β therapy resulted in a significantly higher percentage (Supplemental Figure 1A) and total number (Supplemental Figure 1B) of non Treg CD4^+^ T cells within the TME compared to GVAX with IgG and αTGF-β monotherapy (*p* < 0.05 for all). Therefore, our data suggest that αTGF-β therapy reduces Tregs within the TME in the setting of combinational therapy with both GVAX and αTGF-β.

### TGF-β blockade enhances antitumor effector T cell responses within the TME in a GVAX therapy dependent manner

**Figure 3:**
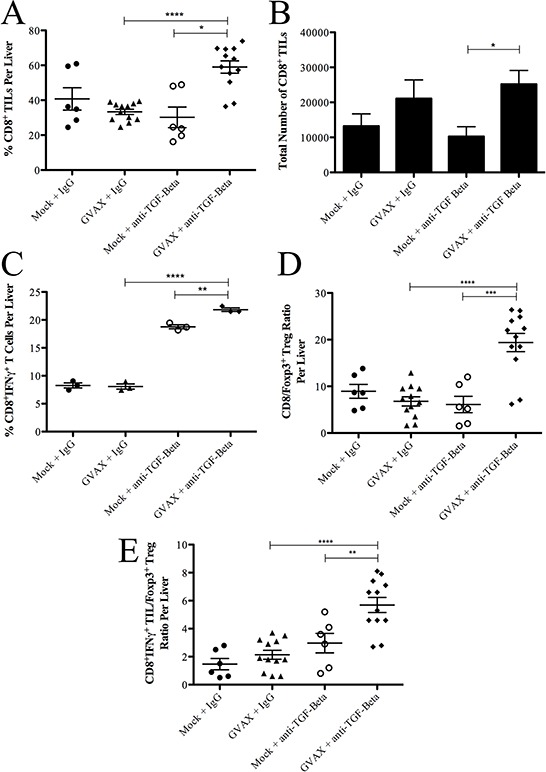
Combination therapy enhances the population of IFNγ^+^ producing CD8^+^ T cell infiltration in the TME in the setting of decreased Tregs **A.** The percentage of CD8^+^ T cell among TILs and **B.** total number of CD8^+^ T cells within the TME. **C.** The percentage of IFNγ^+^ producing CD8^+^ T cells among all CD8^+^ T cells in the TME. The ratio of **D.** CD8^+^ TIL to Tregs and **E.** CD8^+^IFNγ^+^ T cells to Tregs. Each experiment consisted of 3 or 6 mice per group, pooled and analyzed individually in duplicate. Data represent mean ± SEM from one experiment repeated at least twice. *p < 0.05, ^**^p < 0.01, ^***^p < 0.001, ^****^p < 0.0001. TILs, tumor infiltrating lymphocytes. TME, tumor microenvironment. Tregs, regulatory T cells

Next, we examined whether Teffs in the TME are affected by TGF-β blockade. Interestingly, neither GVAX therapy nor αTGF-β therapy changed the percentage of CD8^+^ TILs compared to control treatment (Figure 3A). As previously observed [26], GVAX therapy enhanced the absolute number of CD8^+^ TILs within the TME (Figure 3B), while αTGF-β therapy had no effect on the absolute number of CD8^+^ TILs. However, the combination of GVAX and αTGF-β significantly enhanced both the percentage and absolute number of CD8^+^ TILs. In contrast, the percentage of IFNγ producing cells among CD8^+^ TILs was not changed by GVAX treatment (Figure 3C). However, αTGF-β did significantly increase the percentage of IFNγ producing cells among CD8^+^ TILs as a monotherapy but more in combination with GVAX. Importantly, both the CD8^+^ T cell to Treg ratio and the CD8^+^IFNγ^+^ T cell to Treg ratio within the TME were enhanced by αTGF-β therapy in a GVAX therapy dependent manner (Figure 3D and 3E, respectively).

**Figure 4:**
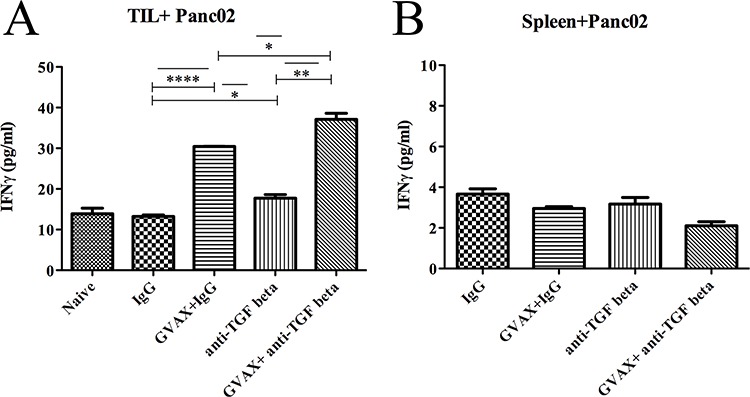
Tumor specific CD8^+^ T cells in the TME is enhanced with combinatorial immunotherapy Irradiated Panc02 tumor cells were used as antigenic targets for CD8^+^ T cells isolated from **A.** TILs and **B.** the spleen. Each experiment consisted of 3 or 6 mice per group, pooled and analyzed individually in duplicate. Data represent mean ± SEM from one experiment repeated at least twice. *p < 0.05, ^**^p < 0.01, ^***^p < 0.001, ^****^p < 0.0001. TILs, tumor infiltrating lymphocytes. TME, tumor microenvironment

To analyze antitumor antigen specific responses, we analyzed the IFNγ production of CD8^+^ TILs incubated with irradiated Panc02 tumor cells (Figure 4A). Both GVAX and αTGF-β therapy enhanced the tumor-specific Teff response as a monotherapy but significantly more as combination therapy. In contrast, tumor-specific Teff response in splenocytes was not enhanced by αTGF-β, GVAX or the combination (Figure 4B).

Taken together, these results suggested that GVAX therapy leads to an increased CD8^+^ T cell presence in the TME; however, it does not enhance the general IFNγ production in these CD8^+^ TILs, in spite of enhancing the tumor-specific IFNγ production. In contrast, αTGF-β therapy does not increase CD8^+^ T cell presence within the TME, but enhances the general IFNγ production of these CD8^+^ TILs. Thus, αTGF-β therapy further enhances the tumor-specific IFNγ production in CD8^+^ TILs in a GVAX therapy dependent manner.

## DISCUSSION

To our knowledge, this is the first demonstration that TGF-β blockade partially depletes Tregs and enhances tumor-specific Teffs in a vaccine dependent manner. Our data show that that the combination of GVAX therapy and TGF-β blockade has direct effects on the tumors by enhancing CD8^+^ T cell infiltration and decreasing the presence of immune suppressive Tregs in the TME. Moreover, enhanced tumor specific CD8^+^ T cell responses were seen within the tumors in mice treated with combinatorial therapy. Importantly, these were not seen in the periphery, implying that the antitumor immune response is activated at the TME level. Finally, combinatorial GVAX and αTGF-β lead to improved clinical responses in this preclinical metastatic pancreatic cancer model.

The findings in this study also validated our previous study [3] showing that the TGF-β pathway is downregulated in the vaccine-induced lymphoid aggregates in the PDAs of patients who had a longer survival following vaccine therapy and also demonstrated enhanced T cell responses to tumor antigens as well as enhanced intratumoral Teff:Treg ratios. Although the role of TGF-β in reducing Tregs in the TME is supported by previous reports, our results also explain why TGF-β blocking agents were not previously found to be very effective as single agents. Our study may identify a more effective way of targeting Tregs by combining the TGF-β blockade with cancer vaccines that recruit both Teffs and Tregs into the TME.

**Figure 5:**
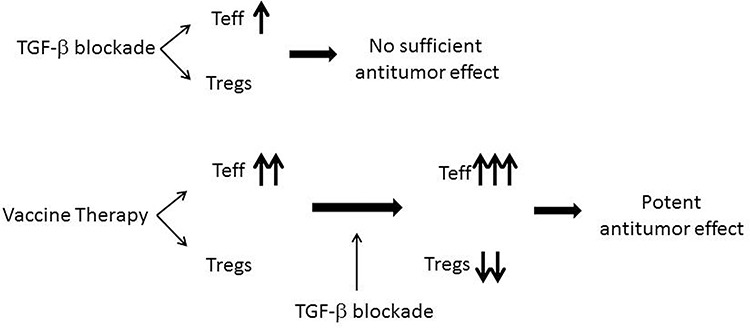
A diagram illustrating the potential effects of TGF-β inhibitors in combination with the pancreatic cancer vaccine therapy

It remains unknown why TGF-β blockade only targets Tregs in the presence of vaccine therapy (Figure 5). It is possible that the signals induced by vaccine therapy coordinate with TGF-β blockade antibodies to target Tregs. More likely, TGF-β blockade only targets vaccine-activated Tregs, but not tumor-residential Tregs. It cannot be excluded that TGF-β blockade directly inhibits the immunosuppressive signaling in vaccine-induced CD8 T cells. The role of TGF-β in Treg development may also lie in the activation of Tregs by the immune response to antigens. One possibility is that TGF-β blockade decreases the Treg number or function and subsequently enhances the effector T cell function in response to the vaccine therapy. Anti-TGF-β monotherapy does have the effect of increasing the percentage of IFNγ producing cells among CD8^+^ TILs as a monotherapy. This is anticipated as published literature shows that TGF-β blockade can activate Teffs through other mechanisms, likely by removing the immunosuppressive signaling secondary to TGF-β acting directly on the Teffs [27, 28]

Targeting Tregs is not sufficient to break the tolerance in the TME in all PDA tumor-bearing mice. The same logic would apply to solely targeting Tregs in human PDA patients. We found only approximately 30% of mice were cured with the combination of TGF-β blockade and GVAX. Similarly, we previously found [26] that the combination of anti-PD-1 antibody and GVAX cured approximately 40% of tumor-bearing mice. Therefore, to maximize the effect of immunotherapy, both PD-1 and Treg pathways should be targeted. In addition, given the pleotropic roles of TGF-β signaling pathways, other immune cell subtypes remain to be examined for a comprehensive understanding of the mechanisms underlying the antitumor effect of the GVAX and TGF-β combination. Moreover, other immunosuppressive pathways remain to be identified and targeted to achieve improved cures that can eventually be tested in patients with PDA.

Our preclinical metastatic PDA model focuses on metastases to the liver, which is the most common metastatic site of human PDA. In particular, the TME in this mouse model reflects that seen in human PDA. Nevertheless, this is still an experimental model as pancreatic cancer patients with liver metastases cannot be cured by the pancreatic cancer GVAX alone [3–4]. The advantage of this model over the autochthonous mouse model is that the tumor formation is timely and spatially controlled, allowing a large sample size in single experiments. Our results suggest that a potentially more effective immunotherapy strategy includes the pancreatic cancer vaccine in combination with TGF-β inhibitor. Immune checkpoint inhibitors such as αPD-1 antibodies may be added to further optimize the immunotherapy strategy once the immune modulating effects of the triple combination of GVAX, TGF-β inhibitor and αPD-1 therapy are further characterized in the preclinical models. Thus, the immunotherapy strategy supported by our preclinical studies warrants testing in human pancreatic cancer clinical trials. Similar immunotherapy strategies may be applicable to other malignancies.

## MATERIALS AND METHODS

### Cell lines and media

Panc02 is highly tumorigenic methylcholanthrene induced pancreatic tumor cell line derived in C57Bl6 mice. [26, 29] KPC tumor cells are a syngeneic pancreatic tumor cell line derived from transgenic mice having tissue-specific Kras and p53 knock-in mutations [30]. B78H1 cells are an MHC class I negative variant of B16 melanoma cell line capable of secreting GM-CSF. All cells were maintained as previously described. [26, 31] Briefly, Panc02 cells were kept in DMEM media (Life Technologies, Frederick, MD), 10% Fetalclone II (ThermoScientific, Rockville, MD), 1% L-glutamine (Life Technologies) and 0.5% penicillin/streptomycin (Life Technologies) at 37°C in 10% CO_2_. KPC cells were maintained in RPMI (Life Technologies), 10% fetal bovine serum (Atlas Biologicals), 1% L-glutamine (Life Technologies), 1% penicillin/streptomycin (Life Technologies), 1% sodium pyruvate (Life Technologies), 1% nonessential amino acids (Life Technologies), and insulin (2 ml) (Novo Nordisk) at 37°C in 5% CO_2_. B78H1 cells were maintained in RPMI media (Life Technologies, Frederick, MD), 10% Fetalclone II (ThermoScientific, Rockville, MD), 1% penicillin/streptomycin (Life Technologies) and 0.5% L-glutamine (Life Technologies) at 37°C in 5% CO_2_.

### Mice and *in vivo* experiments

Mice were purchased from Harlan Laboratories (Frederick, MD) and maintained according to the Institutional Animal Care and Use Committee guidelines. The hemispleen preclinical pancreatic cancer model was performed as previously described [25, 26]. In the Panc02 hemispleen model, tumor inoculation was performed with 2 × 10^6^ cells per 100 μL on day 0. Panc02 GVAX was prepared and administered as previously described [24, 26]. Briefly, 1 × 10^6^ Panc02 cells and 1 × 10^6^ B78H1 per 100 uL were administered subcutaneously in 3 limb nodal basins after irradiation at 50 Gy. Mock vaccine consisted of 1 × 10^6^ B78H1 cells per 100 ul administered subcutaneously in 3 limb nodal basins after irradiation at 50 Gy. Vaccinations were performed on days 4, 11 and 18. One hundred micrograms of mouse αTGF-β (Clone 1D11) (Bio X Cell, West Lebanon, NH) or IgG isotype control (Mouse IgG1) (Bio X Cell) was administered intraperitoneally (IP) three times weekly starting on day 3. Anti-PD-1 (RMP1–14) (Bio X Cell) was given twice weekly (100 ug IP) starting on day 3 for 3 weeks.

The KPC hemispleen model was performed using 1 × 10^5^ tumor cells per 100 uL for each tumor inoculation on day 0. A single allogeneic KPC GVAX vaccination was performed on day 4 with 1 × 10^6^ KPC cells and 1 × 10^6^ B78H1 per 100 uL administered subcutaneously in 3 limb nodal basins after irradiation at 50 Gy. Mouse αTGF-β (Clone 1D11) or IgG isotype control (Mouse IgG1) (Bio X Cell) was administered three times (day 3, 5 and 7) at 100 ug IP per dose.

### Immune analysis of liver-infiltrating lymphocytes and spleen

Immune analysis was performed on day 10 after Panc02 tumor inoculation. Mice received 1 Panc02 GVAX vaccination on day 4 and αTGF-β/IgG on day 3, 5 and 7 (100 ug IP). Tissue processing was performed as previously described [26].

### Cell staining, flow cytometry and intracellular staining

Cell staining, flow cytometry and intracellular staining was performed as previously described. [26] Isolated spleen and liver infiltrating lymphocytes were stained with Live Dead Near-IR Dead Cell kit (Invitrogen), CD3-BV785 (Biolegend), CD45-BV510 (Biolegend), CD8-PE-Cy7 (Biolegend), CD4-PE-CF594 (Becton Dickinson), and CD25-PE-Cy7 (Becton Dickinson). Intracellular staining was performed using anti-mouse forkhead box P3 (FoxP3)-AF488 (MF23; BD Pharmingen). Intracellular cell staining for IFNγ and flow cytometry was performed as previously described using IFNγ-BV421 (Biolegend) [26].

### Mouse IFN-γ enzyme-linked immunosorbent assay (ELISA)

CD8^+^ T cells from liver infiltrating lymphocytes and splenocytes were isolated using CD8 negative isolation kits (Life Technologies) according to the manufacturer's protocol. Irradiated Panc02 tumor cells were added to isolated CD8 T cells at a ratio of 5:1 (2 × 10^5^ CD8^+^ T cells combined with 4 × 10^4^ Panc02 tumor cells) and were subsequently incubated for 18 hours in 5% CO_2_ at 37°C. The ELISA assay was then conducted using mouse IFNγ ELISA Ready-SET-Go assay per the manufacturer's protocol (eBioscience).

### Statistical analysis

The cure rates are calculated as the percentages of mice that remain free of tumor according to the necropsy examination at the end of the experiment (Day 90 following the tumor implantation). Statistical analysis for comparison of cure rates were evaluated using *χ*^2^ test. Mean values between groups for cell number, percentage and cytokine expression were evaluated using an unpaired Student's *t* test. *P* < 0.05 was considered statistically significant.

## SUPPLEMENTARY FIGURE


