# Supplementary material for: TGF-β blockade depletes T regulatory cells from metastatic pancreatic tumors in a vaccine dependent manner
Source: Oncotarget. 2015 Oct 15;6(40):43005–15. doi: 10.18632/oncotarget.5656 (PMC4767487; doi:10.18632/oncotarget.5656)
Supplement: Supplementary file 1 [file oncotarget-06-43005-s001.pdf]

SUPPLEMENTARY FIGURE

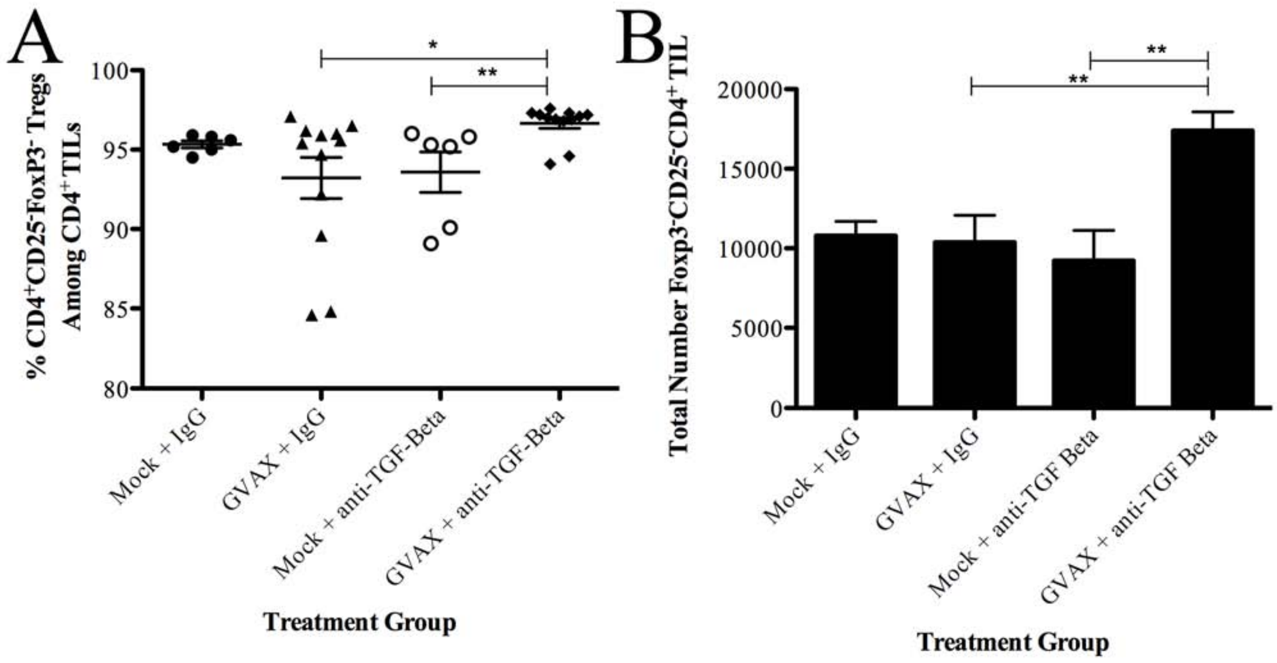

Supplementary Figure S1: Combination therapy with GVAX and αTGF-β increases $CD25^-$ $Foxp3^-$ $CD4^+$ T cell presence in PDA in TME. A. The percentage of $CD25^-$ $Foxp3^-$ $CD4^+$ T cells among total lymphocytes and **B.** the total number of $CD25^-$ $Foxp3^-$ $CD4^+$ TILs after Panc02 hemispleen and the indicated therapy. Each experiment consisted of 3 or 6 mice per group, pooled and analyzed individually in duplicate. Data represent mean ± SEM from one experiment repeated at least twice. $^*p < 0.05$, $^{**}p < 0.01$. TILs, tumor infiltrating lymphocytes. PDA, pancreatic ductal adenocarcinoma. TME, tumor microenvironment.
